# Treatment Options and Post-Treatment Malignant Transformation Rate of Actinic Cheilitis: A Systematic Review

**DOI:** 10.3390/cancers13133354

**Published:** 2021-07-04

**Authors:** Katerina Bakirtzi, Ilias Papadimitriou, Dimitrios Andreadis, Elena Sotiriou

**Affiliations:** 1 First Department of Dermatology and Venereology, School of Medicine, Aristotle University of Thessaloniki, P.O. Box 54643 Thessaloniki, Greece; mpakirtzi@auth.gr (K.B.); ipapadimi@auth.gr (I.P.); 2 Department of Oral Medicine and Pathology, School of Dentistry, Aristotle University of Thessaloniki, P.O. Box 54624 Thessaloniki, Greece; dandrea@dent.auth.gr

**Keywords:** actinic cheilitis, treatment, imiquimod, photodynamic therapy, vermilionectomy, laser, diclofenac

## Abstract

**Simple Summary:**

Actinic cheilitis is a precancerous condition that may evolve to a more aggressive type of skin cancer. Therefore, its therapy is crucial for the disease prognosis. In this systematic review, we tried to identify the best therapies of actinic cheilitis regarding safety, efficacy, recurrences, and the potential to progress to skin cancer. The therapeutic approach comprised invasive and topical treatments. The invasive therapies, such as partial surgery and laser treatments, had the best cosmetic and therapeutic results with few recurrences. Photodynamic therapy demonstrated satisfactory outcomes, while topical treatments were the least beneficial. Notably, the efficacy of photodynamic therapy was improved when combined with 5% imiquimod. However, except from photodynamic therapy, the other modalities were assessed in a limited number of patients. Finally, when actinic cheilitis is treated, no risk of cancer progression exists. Larger studies are necessary to confirm these results.

**Abstract:**

Actinic cheilitis is a premalignant condition that may evolve to squamous cell carcinoma. A consensus on its management has not been established, and large clinical trials are lacking. We aimed to review the existing data regarding the treatment of actinic cheilitis with various modalities regarding safety, efficacy, recursions, and post-treatment malignant transformation. A systematic review was conducted through Pubmed, Ovid and the Cochrane library for studies in English language and the references of included papers from inception to January 2021. Case series were considered if ≥6 patients were included. Of the 698 articles, 36 studies and, overall, 699 patients were eventually reviewed. Laser ablation and vermilionectomy provided the best clinical and aesthetic outcomes with few recurrences, while photodynamic therapy was linked to more relapses. Generally, the adverse events were minor and there was no risk of post-treatment malignant transformation. The limitations of our review include the heterogeneity and the small number of patients across studies. Conclusively, invasive treatments demonstrated superior therapeutic and safety profile. Nevertheless, high-quality head-to-head studies that assess different modalities for actinic cheilitis and report patient preferences are lacking.

## 1. Introduction

Actinic cheilitis (AC) is a premalignant lesion on the lips in patients who are overexposed to sunlight, and it has a significant chance of evolving into invasive squamous cell carcinoma (SCC). It primarily affects the lower lip of male individuals over the age of 50, and its clinical features include dryness, atrophy, scaling, erythema, ulceration, and a poorly demarcated border [1,2,3,4]. Most patients are of the Fitzpatrick I-II skin phototype [3]. It is also observed that people of lower education level and poor lifestyle conditions are more likely to develop AC [5]. The dermoscopic characteristics of AC comprise white structureless areas, scales, white halos of the vermilion of the lip, and erosions [6,7]. The slow progression of AC usually leads to a delay in the diagnosis, as it is often mistakenly regarded as a regular feature of aging [8].

The rate of malignant transformation of AC into SCC varies between 10 and 30%, while it is reported that 95% of SCCs on the lip occur on the ground of preexisting ACs [9,10]. Moreover, whereas up to 6% of cutaneous SCCs metastasize, the metastasis rate for a SCC located on the lips is four times higher than its peripheral counterpart [11]. Therefore, early detection and treatment of AC are of great value, since they could largely prevent SCC development.

Nevertheless, to date, no consensus on the proper management of AC exists. Surgical removal techniques exist, with vermilionectomy being the most commonly employed [12]. Yet, the surgical approach is destructive and is linked to various complications, such as scarring, persistent oedema and anaesthesia [13]. Conventional therapeutic approaches include topical chemo- or immunotherapy and radiation-based treatment, with the former being the less effective due to low patient adherence [14]. Topical application of fluorouracil FU, 5% imiquimod (5% IMI) and 3% diclofenac in 2.5% hyaluronic acid (DHA), 0.015% ingenol mebutate (IngMeb) and trichloroacetic acid (TCA), regarding topical regimens, photodynamic therapy (PDT) and CO_2_ laser ablation (CO_2_L), with or without aminolevulinic acid (ALA), or methyl-aminolevulinic acid (MAL), cryosurgery (CRYO) and electrodessication (ELD,), regarding radiation-based and minimally invasive treatments, have been described [15]. Although there is some literature on the efficacy of AC treatments, there is still a lack of high-quality research to direct appropriate management decisions. The present systematic review aims to offer an overview of the efficacy of the current AC treatments with respect to clinical responses and, where available, histopathological or dermoscopic clearance, recurrence rates and the rate of post-treatment AC malignant transformation.

## 2. Search Strategy

This systematic review was conducted according to the Meta-analysis of Observational Studies in Epidemiology (MOOSE) proposal and the Preferred Reporting Items for Systematic Reviews and Meta-Analyses PRISMA guidelines [16,17]. The research was performed in MEDLINE through PubMed, Embase through Ovid and in the Cochrane Central Register of Controlled Trials (CENTRAL) library. The references of the suitable papers were screened for further relevant publications. A forward search was considered excessive since the searches already carried out yielded a plethora of results. The search was conducted in January 2021.

The search protocol was ((“actinic” OR “solar”) AND (“cheilitis”) OR (“cheilosis”) AND (“treatment”)) examining both medical subject headings (MeSH) and free text. Eligible studies were clinical trials, prospective and retrospective studies on human subjects and case series of at least six patients written in the English language. All studies needed to include cases with a pathological AC diagnosis, having received either surgical or non-invasive treatment, and report the clinical and/or pathological response as their outcome. Cross-sectional studies with no post-treatment follow-up visits were also excluded. Studies were also ruled out if they did not contain the results of AC therapies or did not fulfill the inclusion criteria.

## 3. Data Extraction

Two blinded reviewers (B.K. and P.I.) extracted data independently based on a standardized extraction form. Any discordance was resolved by consensus or by the involvement of a third investigator who was experienced in performing systematic reviews and meta-analyses (L.A.). The following data were collected from each eligible paper: year of the study, authors, publication date, type of manuscript, title, country, the treatment being studied (type, duration), study design, study population (sample size, age, sex, risk factors and comorbidities), means of diagnosis (histopathological or clinical), and sponsorship reported. Treatment evaluation was based on treatment response rate, healing time, recurrence rate, side effects, follow-up time, follow-up biopsy and aesthetic results. The risk of bias in every eligible study was classified as “low”, “moderate”, or “high” by the same reviewers per the PRISMA guidelines.

## 4. Results

The database search yielded 698 articles. After duplicates were removed, 281 articles were identified and, when titles and abstracts were reviewed, 91 papers were subsequently ruled out since they did not satisfy the inclusion and exclusion criteria. The 190 remaining articles were thoroughly reassessed for eligibility. After exclusion criteria were applied, 58 research papers remained. Comprehensive data extraction was performed for these 58 papers. Twenty-two studies were excluded based on the quality criteria, eventually leaving 36 papers for the data analysis, published between 1977 and 2019 [13,18,19,20,21,22,23,24,25,26,27,28,29,30,31,32,33,34,35,36,37,38,39,40,41,42,43,44,45,46,47,48,49,50,51,52]. High risk of bias levels was detected in all the included studies.

The shortlisted studies consisted of two RCTs, six retrospective and 22 prospective studies, and three case series. The number of patients in each study varied between six and fifty-two (699 in total) (Table 1). The gender was reported in 678 patients, most of whom were male (75.07%; 509/678), and the patients’ ages ranged from 26 to 93 (mean: 63.18 ± 9.3). The localization of AC was recorded in 573 patients, with the lower lip being the most predominant site in 556 cases (97.03%), and the upper lip (1.05%; 6/573) or both lips (1.75%; 10/573) being significantly less affected. In nine studies, the percentage of the lip surface area affected by AC was also reported: >75% involvement in 24.77% of the patients (55/222), while in 70.72% (157/222) of patients, the lip involvement ranged between 50% and 75%. Data regarding the risk factors for AC development of the study population were collected from 30 articles. Seventy-two patients had fair skin, sixty-four had positive smoking and twenty-four positive alcohol drinking history, fifty-eight had suffered from skin tumors (non-melanoma skin cancer or melanoma: 49 patients; previous SCC of the lower lip: 9 patients), thirty-six were outdoor workers, four reported intense sunlight exposure and one was immunosuppressed.

There is not a widely accepted clinical measurement tool for the severity and therapeutic outcomes for AC to date. These outcome measures used in the studies were comparable to the newly established core outcome set for actinic keratoses clinical trials [54]. Most articles presented results regarding the clearance—based on the clinical or histopathologic response, recurrence or progression rate, side effects, long-term follow-up, cosmetic outcome and patient satisfaction—and some reported on treatment discontinuation, patient adherence or healing time (Table 2).

### 4.1. Therapies for AC

Overall, 699 patients have been treated with the following therapies: laser-therapy (319; 45.64%) [13,23,24,25,26,29,30,37,46,50,51,52], PDT (241; 34.48%) [18,20,31,32,34,35,36,38,39,41,42,43,44,45], DHA (62; 8.87%) [19,22,38,53], MAL PDT + 5% IMI (34; 4.86%) [33], MAL or ALA plus laser (laser-PDT) (33; 4.72%) [31], 5% IMI (25; 3.58%) [19,47], FU (28; 4.0%) [23,48,49], partial surgery (28, 4.0%) [23,29,50], 0.015% IngMeb (17; 2.43%) [19,40], 50% TCA (10; 1.43%) [23] and ALA-PDT plus excimer dye laser (1; 0.14%) [30]. Of the shortlisted articles, 31 investigated one therapy alone, treating 533 patients in total [18,20,22,24,25,26,32,33,35,36,37,38,39,40,41,42,43,44,45,46,47,48,49,50,51,52,53]. In four articles [13,29,30,31], the efficacy of two modalities has been compared in 198 treated areas of 142 patients: one study compared laser-PDT (erbium-doped yttrium aluminium garnet (Er:YAG) and MAL-PDT) to MAL PDT alone [31], one study compared two different methods of CO_2_ laser [13], one study compared dye laser to laser and ALA-PDT [30], and one study compared CO_2_ laser to ELD with high energy [29]. Only one paper (30 patients) compared the efficacy of three different therapies (5% IMI vs. 0.015% IngMeb vs. DHA) [19] and only one study (40 patients) compared the efficacy of four therapeutic approaches (5% FU vs. 50% TCA vs. CO_2_ laser vs. lip shave) [23].

### 4.2. Outcomes

It must be noted at the outset that all treatment options included a limited number of cases, except for studies regarding PDT and laser therapy. Furthermore, outcomes were often evaluated only on a certain number of individuals and not on the whole study population who underwent each treatment.

### 4.3. Therapeutic Response

Thirty-one out of 36 studies reported complete clinical clearance of the treated area: a rate of 85.93% of patients [13,18,19,20,22,23,24,25,26,30,31,32,33,34,35,36,38,39,40,41,42,43,45,46,47,48,49,50,51,53]. Partial clinical response was observed in 25.37% of cases. Poor treatment effect and clinical deterioration were only described in a limited number of patients. The overall clinical recurrence rate, as estimated in 21 articles [13,20,22,23,24,25,30,31,32,33,38,39,41,42,45,46,48,50,51], was 11.24% of the treated areas. In terms of the histopathologic outcomes, a post-treatment biopsy was performed in 23 studies [13,18,19,20,23,24,25,26,29,30,31,32,33,34,35,36,41,44,47,49,50,51,53]. In 88.43% of the treated areas that were biopsied, evidence of complete response was reported, with 64.07% obtaining complete clearance.

### 4.4. Invasive Treatments

All laser treatments demonstrated excellent efficacy, with 93.39% (226/242) of patients included in the studies achieving complete clinical response, varying from 93.04% (214/230) for CO_2_ laser ablation to 100% for Er: YAG (12/12) [13,23,24,25,26,29,30,37,46,50,52]. Complete histopathological response was reported in 96% of patients (72/75), whereas the recurrence rate was 6.42% (14/218). The number of AEs per patient treated with laser therapy was less than one (0.42/case), and the aesthetic result was deemed exceptional in 100% of cases. All patients who underwent partial surgery obtained complete response (14/14, 100%) and did not report any recurrences [23,29,50]. More than 80% of patients with histopathological evaluation achieved the relevant complete response (10/12; 83.33%), and the number of adverse events per patient was minimal (0.3/case).

### 4.5. Non-Invasive Treatments

An almost 80% (27/34; 79.41%) complete clinical response was achieved among patients treated with MAL-PDT and 5% IMI, whereas complete histopathological response reached 64.71% (22/34). The recurrence rate was 5.88% (2/34), and the number of adverse events per patient was 5.4, with two patients (5.88%) discontinuing treatment due to side effects [33]. Regarding laser application in conjunction with PDT, the relevant studies reported complete clinical response in 75.76% (25/33) of patients, varying between 68.4% for ALA PDT-dye laser and 85.7% for MAL PDT-Er: YAG laser, while the clinical recurrence rate was calculated at 6.1% (2/33) [30,31]. When FU was applied, complete clinical response was obtained in a satisfactory percentage of patients (21/28; 75.0%), with 1% FU demonstrating excellent performance (100% complete response) compared to 5% FU (68.21%); the recurrence rate, however, was quite high (7/22; 31.8%). In the histopathological follow-up evaluation, 5/6 (83.33%) patients achieved partial clearance, and 1/6 (16.67%) reported poor response. Treatment discontinuation due to adverse events was noted in 10.0% of patients [23,48,49]. During 5% IMI therapy, complete clinical response was experienced by 76.0% (19/25) of subjects, while the number of AEs per patient was calculated at 3.1 [19,47]. Photodynamic therapy was a treatment option that was thoroughly investigated. A total of 148 cases out of 222 (66.67%) across the included studies achieved complete clinical response. The MAL daylight treatment outperformed all other approaches (82.63%), whereas methyl-aminoxopentanoate-PDT had the lowest scores (55.6%). Furthermore, PDT with aminolevulinic acid rather than with methyl-aminolevulinate produced better clinical results (73.49% vs. 63.81% complete response rate, respectively). Complete histopathological response was obtained in 49.48% (48/97) of patients. Reflecting the clinical outcomes, ALA-PDT performed better than MAL-PDT (53.24% and 23.41%, respectively) in the histopathological evaluation. The cumulative recurrence rate of the relevant studies was calculated to be 14.07% (19/135). The number of side effects per patient was estimated to be 2.4, whereas the treatment interruption due to AEs was reported in 5.86% (13/222) of patients. Excellent cosmetic results were recorded in 92 out of 136 (67.65%) subjects [18,20,31,32,34,35,36,39,41,42,43,44,45]. The complete response rate was 41.18% (7/17) through ingenol mebutate therapy. No recurrences were observed throughout the follow-up period. All patients experienced adverse events; however, none of them discontinued treatment, as the side effects reported were mild and resolved within a maximum of two weeks without any medical intervention [19,40]. When patients were treated with DHA, almost half of them (28/62; 45.16%) obtained complete clinical response, whereas complete histopathological response was observed in 66.67% (4/6) of the assessed cases. Regarding the aesthetic results, all respondents (6/6) rated the outcomes as excellent. The recurrence rate was estimated in 6.52% (3/46) of cases, whereas seven patients discontinued treatment due to AEs (15.22%) [19,22,38,53]. Finally, after 50% TCA application, only 30% of cases (3/10) achieved complete clinical clearance [23].

### 4.6. Cosmetic Outcome

Seventeen articles [24,25,26,27,28,30,31,32,34,35,36,39,43,45,51,53,55] assessed the cosmetic result, which was described as excellent in 74.63% of patients. The cosmetic outcome depicted the patients’ perspective in two studies [28,36] and the physician’s evaluation in 14 studies [24,25,26,27,30,31,32,34,35,39,43,45,51,53,55], while, in one study, both measurements were used [34]. All patients treated with laser ablation or DHA reported excellent aesthetic results with no scarring. In a study of two surgical techniques, W-plasty outperformed vermilionectomy in terms of scar retraction [55]. Excellent cosmetic outcomes ranged from 58% to 88% in patients who underwent vermilionectomy [27,28]. The relevant PDT results varied widely: one study reported excellent results in 60% of cases, while fair or poor outcomes were observed in 40% of cases [31], two papers reported excellent outcomes in nearly 80% of cases [32,34] and another study reported very good results in 33% of patients [36].

### 4.7. Healing Time in Different Studies

The mean healing time estimated in 13 articles [13,18,24,25,26,29,30,37,49,50,51,52] was 2.8 weeks (Range: 0.4–4 weeks). The healing time was primarily reported for CO_2_ laser therapy, followed by MAL-PDT, and was reported less for 5% IMI, 1% FU, ELD, dermabrasion, a combination of dye-laser and ALA-PDT, and for Er: YAG laser.

### 4.8. Adverse Events

Twenty-four papers provided data on AEs [13,18,19,20,26,27,31,32,33,34,35,36,37,39,40,43,44,45,46,47,50,51,53] with a total of 1027 AEs experienced by 541 patients. The most common side effects included erythema, pain, edema and burning sensation. Moreover, based on information provided by three studies, a mean VAS pain value of 5.62 ± 1.75 in 69 patients was calculated (57 treated with PDT, 12 with Er: YAG) [26,35,44]. Overall, 16 patients discontinued treatment due to AEs, as reported in six studies [22,23,33,35,38,43]. Most AEs were mild to moderate in severity and subsided within two weeks post-treatment without therapeutic intervention [34,53]. Persistent AEs for up to one year were reported after surgical treatment and included labial tension and diminished sensitivity in nearly 36% of cases [27].

### 4.9. Post-Treatment Malignant Transformation

Malignant transformation after surgical treatment was examined in three longitudinal studies, and none of them reported any incidence [13,23,25]. Nevertheless, in a study where different approaches of CO_2_ laser implementation were compared and in a case series of CO_2_ vermilionectomy, low rates (1/43; 2.33% and 1/14; 7.14%) of malignant transformation in the treated areas were observed [29,37]. On the other hand, neither of the two reports [23,34] where patients had undergone non-invasive treatment for AC with long follow-up periods (1.5–4 years) could detect any case of post-treatment malignant transformation.

### 4.10. Assessment of Recurrence

Five articles reported the effect of laser ablation (four regarding CO_2_ laser and one regarding Er: YAG laser ablation) [13,23,24,25,26]. Of those, in only one study of 40 cases, the recurrence rate reached 13% [13]. On the other hand, no recurrence was observed through vermilionectomy in any of the relevant studies [27,28,55]. Topical treatments presented a high recurrence rate with diclofenac 3% gel obtaining 33% [53] and FU application 55% [23] recurrence rates. Both studies regarding laser treatment combined with PDT presented recurrence rates of 8% [30,31]. Six papers presented the results of PDT monotherapy [18,31,32,34,35,36], with one those presenting the results of PDT combined with 5% IMI [33]; the latter achieved the lowest recurrence rate (12%). Although, in two papers on PDT monotherapy, no recurrences have been observed, in the remaining four articles, 25–60% of patients suffered from AC recurrence. Three randomized controlled trials compared the outcomes of various therapies. The first compared topical FU application, chemical peel, vermilionectomy and CO_2_ laser ablation [23]. Among the four modalities, chemical peel obtained the highest recurrence rate, which reached 70%, while patients treated with CO_2_ laser ablation or vermilionectomy did not experience any recurrence. The second RCT compared laser-assisted PDT to PDT alone, and it was proved that laser-assisted PDT outperformed PDT monotherapy in terms of recurrence rates (8% vs. 50%, respectively) [31]. Lastly, the third RCT suggested that W-plasty compared to classic vermilionectomy were equally satisfying when recurrence rates were concerned [55].

## 5. Risk of Bias and Quality of the Shortlisted Studies

The risk of bias in individual papers was determined as per the Cochrane Reviews recommendations using the updated RoB-2 tool [56]. As stated in the Cochrane handbook, “a bias is a systematic error, or deviation from the truth, in results or inferences, which means that multiple replications of the same study would reach the wrong answer on average”. Six kinds of bias were assessed, namely: 1. Selection bias, when the study population does not represent the target population. 2. Performance bias, when the conduct of a study negligently introduces differences between randomized groups other than the intervention being investigated. 3. Attrition bias, when subjects are lost to follow-up, or they miss at least one measurement time points during the study period. 4. Reporting bias, when a trial reports only part of its estimated outcomes. 5. Other sources of bias. 6. Overall.

Bias analysis showed that all articles included in the review presented a high risk of bias. Only two studies’ designs proceeded to patient randomization [13,31]. None of the studies followed the blinding process either for therapeutic intervention or evaluation. The follow-up time varied between 3 and 48 months across studies. All articles reporting a follow-up time of fewer than 8 months had histopathological verification of AC clearance. Most trials were non-randomized, observational cohort studies. Even though the number of randomized clinical trials was fairly limited, the data obtained from these papers suggest that laser ablation or vermilionectomy demonstrate the lowest recurrence rates. Finally, given the small number of trials testing each therapeutic approach and the heterogeneity of results and study design, statistical comparison or a meta-analysis was deemed inappropriate.

## 6. Discussion

In this systematic review, we demonstrated the results of 36 studies of AC treatment and assessed the relevant outcomes of the surgical (laser ablation and vermilionectomy) and topical therapeutic (5-FU, diclofenac gel and PDT) approach. We concluded that the best response was obtained through partial surgery and laser therapy, either alone or combined with PDT, with MAL PDT + 5% IMI, FU and PDT alone achieving lower clearance rates. Our findings derived from pool data analysis and individual studies whenever the number of studies for each aspect was not enough to accrue meaningful conclusions.

A recent consensus established an international core outcome set for clinical trials on actinic keratosis treatment based on physician and patient Delphi surveys [54]. The significance of the aesthetic result and adverse events were ranked, among other factors, lower by patients compared to physicians. This could be explained by the composition of the study population, which included patients with a history of skin cancer; therefore, recurrence was inevitably deemed more important than the cosmetic outcome. Furthermore, the localization of the actinic keratoses was not determined in the consensus. It may thus be possible for cosmetic outcomes to be of particular relevance for patients with AC due to the visibility of the lesions.

It should be noted that, so far, the studies on AC do not concentrate on patients’ future treatment preferences; this is made apparent in the available literature. Only one study investigated patient treatment satisfaction, with 80% of cases considering the treatment as beneficial [21]. Patient satisfaction should be more closely examined, at least regarding surgical versus topical treatment, considering the invasive nature of vermilionectomy and laser-assisted therapies.

Even though the number of patients for each treatment was low in each study, the cumulative number of areas treated with each therapy was significant. Specifically, at least 200 cases were treated with laser therapies and PTD alone, and sixty-two areas with DHA. Laser therapy—mainly CO_2_ laser treatment—outperformed the other therapeutic options in all aspects, including high rates of complete response and low recurrence rates. Carbon dioxide laser ablation seems to be linked to fewer side effects with a shorter time to resolution than vermilionectomy, although head-to-head trials are lacking [23,55]. Unlike non- or minimally invasive treatments, though, vermilionectomy has the added benefit of enabling the histopathological evaluation of the lesion. Nοvel surgical procedures such as W-plasty may provide comparable results to conventional vermilionectomy with better cosmetic outcomes. Our results regarding the beneficial effect of CO_2_ laser therapy for actinic cheilitis are in line with the guidelines for the SCC from the National Comprehensive Cancer Network of the U.S.A., which suggest ablative laser vermilionectomy as a recommended treatment for extensive AC [57]. On the other hand, there are no relevant European and British guidelines for the management of AC.

Concerning other therapeutic options, PDT scored relatively lower in terms of complete response than other treatments such a laser therapy, FU and 5% IMI, while over 12% of patients suffered from a recurrence. Moreover, DHA showed an exceptionally low complete response rate, whereas the recurrence rate is as good as laser therapy. Both treatments seem to be less efficient, possibly due to crusts which may impede the therapeutic effect and patient compliance.

As regards the other therapies, the relevant results should be interpreted with caution, given the small study population of each paper. On the whole, the subsequent employment of two different modalities seems to have a synergistic effect on the therapeutic outcome of each treatment. By way of example, unlike the application of 5% IMI and MAL-PDT alone, which were characterized by modest results, the combination of MAL-PDT with 5% IMI yielded an almost 80% complete response. One possible explanation for this superior efficacy may lie in their different modes of action, since PDT selectively destroys cancer cells and IMI enhances the immune response. Sotiriou et al. suggested that the inflammation generated after PDT could promote the activation of innate immunity against the malignant cells through the action of 5% IMI [33]. Finally, the different evaluation criteria of the histopathologic response across studies and the post-treatment biopsies at different time points do not permit a valid clinicopathological correlation of the treatment outcome. Nevertheless, most therapies provided a satisfactory safety profile with few side effects and excellent aesthetic results.

Minimally invasive therapies have the additional advantage of higher patient compliance rates, unlike topical treatments, which are more prone to discontinuation. More sophisticated procedures that minimize side effects may make the surgical approach a more attractive and beneficial option given its high efficacy. Therefore, it would be recommended that the risk–benefit ratio should be assessed for each of the therapeutic modalities during the treatment decision process, offering tailored management. In high-risk patients, for example, such as immunosuppressed patients or patients with a history of skin cancer, where definite and timely cures are essential, the surgical approach should be preferrable over topical treatment. Nevertheless, in younger, low-risk individuals, non-invasive therapies may be more appropriate. Patients’ preferences and well-being should also be considered before making management decisions.

It should be remembered that the original purpose of AC therapy is to minimize the risk of the pre-malignant lesions evolving into a squamous cell carcinoma in the future. Most SCC cases reported in a study were diagnosed clinically, even when the diagnostic confidence was low [46]. Therefore, in these cases, the malignant lesion could pre-exist prior to treatment initiation. The other case series describing the malignant transformation of AC included patients with mostly moderate and severe epithelial dysplasia [29,37]. A possible explanation could be that the features of epithelial dysplasia create the conditions for potential malignancy. Whatever the case, well-designed studies exploring the malignization incidence are essential to provide accurate data for the various treatments, especially for topical therapies.

The results of our review should be viewed in light of some limitations. First, the sample size of most studies was small, while the substantial diversity in the quality and characteristics of presented evidence did not allow any direct comparison. Moreover, it should be noted that the data provided by the relevant studies were restricted only to positive outcomes, and the negative or ambiguous results of AC therapies could be ruled out. Third, there was heterogeneity in the histopathologic post-treatment evaluation and follow-up time points across the published studies. Lastly, none of the studies assessed cryotherapy as a therapeutic option, a treatment that has become a mainstay for the management of AC in everyday clinical practice.

## 7. Conclusions

Our review highlights the need for higher quality and more comprehensive studies in the field of AC management. However, given the available data, our review suggests that laser treatment alone or combined with PDT seems to offer the best clinical outcomes, while FU, 5% IMI and PDT alone or combined have a satisfactory therapeutic profile. Large, randomized controlled studies are necessary to validate the kinds of conclusions drawn from this review in terms of the efficacy and safety of the traditional therapies for AC so that dermatologists can select the optimum therapeutic approach for these patients.

## Figures and Tables

**Table 1:** Studies and therapeutic process per treatment modality.

Treatment	Number of Studies	Number of Patients	Specific Treatment	Days of Treatment	Follow-Up Time (Range in Months)
Partial surgery [23,29,50]	3	28	CO_2_ laser ablation, Electrodessication vs. CO_2_ laser, Chemical peel	1	3–48
Laser [13,23,24,25,26,29,30,37,46,50,51,52]	12	278	CO_2_ laser, Er: YAG laser	1–3	1.3–60
MAL PDT + 5%IMI [33]	1	34	-	1	12
Laser-mediated PDT [30,31]	2	33	ALA-dye, MAL-Er: YAG	1–3	1–12
FU [23,49,51]	3	28	1% and 5% fluorouracil	12–21	2–48
5% IMI [19,47]	2	25	-	12–30	1–18
PDT [18,20,31,32,34,35,36,39,41,42,43,44,45]	13	241	Daylight PDT, ALA PDT, MAL PDT	1–6	1–60
0.015% IngMeb [19,40]	2	17	-	1	1–10
50%TCA [23]	1	10	-	1	48
DHA [19,22,38,53]	4	62	-	1	1–6

MAL—methyl-aminolevulinic acid; ALA—aminolevulinic acid; Er: Yag—Erbium: YAG; IMI—imiquimod; PDT—photodynamic therapy; FU—fluorouracil; DHA—3% diclofenac in 2.5% hyaluronic acid; TCA—trichloroacetic acid; IngMeb—ingenol mebutate.

**Table 2:** Study results per treatment modality.

Treatment	Complete Response Rate (%)	Recurrence Rate (%)	Adverse Events	Excellent Cosmetic Results (%)	Discontinuation Rate (%)
Partial surgery [23,29,50]	100	0.0	Scarring	N/A	N/A
Laser [13,23,24,25,26,29,30,37,46,50,51,52]	93.39	6.42	Scarring, pain, oedema, erosion, pruritus	100.0	N/A
MAL PDT + 5%IMI [33]	79.41	5.88	Pain, erythema, burning sensation, scarring, oedema, pruritus, rash	N/A	5.88
Laser-mediated PDT [30,31]	75.76	6.10	Erythema, burning sensation, oedema, erosions	N/A	N/A
FU [23,49,51]	75.0	31.80	N/A	N/A	10.0
5% IMI [19,47]	76.0	N/A	Erythema, oedema, induration, erosions, burning sensation	N/A	N/A
PDT [18,20,31,32,34,35,36,39,41,42,43,44,45]	66.67	14.07	Pain, erythema, oedema, scaling, rash, erosions, burning sensation, scarring	67.65	5.86
0.015% IngMeb [19,40]	41.18	0.0	Erythema, oedema, scaling, erosions, burning sensation	N/A	0.0
50%TCA [23]	30.0	70.0	N/A	N/A	N/A
DHA [19,22,38,53]	45.16	6.52	Erythema, oedema, burning sensation	100.0	15.22

N/A—not available; MAL—methyl-aminolevulinic acid; ALA—aminolevulinic acid; Er: Yag—Erbium: YAG; IMI—imiquimod; PDT—photodynamic therapy; FU—fluorouracil; DHA—3% diclofenac in 2.5% hyaluronic acid; TCA—trichloroacetic acid; IngMeb—ingenol mebutate.
